# Attitude of Syrian students toward GAD patients: An online cross-sectional study

**DOI:** 10.3389/fpubh.2022.955321

**Published:** 2022-11-09

**Authors:** Sarya Swed, Sheikh Shoib, Ubaid Khan, Amro A. El-Sakka, Mohammad Badr Almoshantaf, Noheir Ashraf Ibrahem Fathy Hassan, Lina Taha Khairy, Agyad Bakkour, Ali Hadi Hussein Muwaili, Karam R. Motawea, Fatima Abubaker Abdalla Abdelmajid, Eman Mohammed Sharif Ahmad, Safaa Mohamed Alsharief Ahmed, Mohammad Mehedi Hasan, Bisher Sawaf, Mhd Kutaiba Albuni, Elias Battikh, Asmaa Zainabo, Hidar Alibrahim, Hazem S. Ghaith, Nashaat Kamal Hamdy Elkalagi

**Affiliations:** ^1^Faculty of Medicine, Aleppo University, Aleppo, Syria; ^2^Department of Psychiatry, Jawahar Lal Nehru Memorial Hospital, Srinagar, Kashmir, India; ^3^Faculty of Medicine, King Edward Medical University Lahore, Lahore, Pakistan; ^4^Faculty of Medicine, Suez Canal University, Ismailia, Egypt; ^5^Department of Neurosurgery, Ibn Al-Nafees Hospital, Damascus, Syria; ^6^Faculty of Medicine, Aswan University, Aswan, Egypt; ^7^Faculty of Medicine, The National Ribat University, Khartoum, Sudan; ^8^Faculty of Medicine, Albaath University, Homs, Syria; ^9^Faculty of Medicine, Ivano-Frankivsk National Medical University, Ivano-Frankivsk, Ukraine; ^10^Faculty of Medicine, Alexandria University, Alexandria, Egypt; ^11^Faculty of Medicine, University of Medical Sciences and Technology, Khartoum, Sudan; ^12^Faculty of Medicine, Nile Valley University, Atbara, Sudan; ^13^Faculty of Medicine, Shendi University, Shendi, Sudan; ^14^Department of Biochemistry and Molecular Biology, Faculty of Life Science, Mawlana Bhashani Science and Technology University, Tangail, Bangladesh; ^15^Department of Internal Medicine, Syrian Private University, Damascus, Syria; ^16^Faculty of Medicine, Damascus University, Damascus, Syria; ^17^Faculty of Medicine, Tishreen University, Lattakia, Syria; ^18^Faculty of Medicine, Al-Azhar University, Cairo, Egypt; ^19^Lecturer in Internal Medicine and Tropical Medicine at Faculty of Medicine Al Arish University, Alarish, Egypt

**Keywords:** GAD (general anxiety disorders), stigma, national, Syria, students

## Abstract

Generalized Anxiety Disorder (GAD) is a prevalent condition and a significant cause of mental disability and poor quality of life. People with GAD have chronic worrying, restlessness, and discrimination from the general public; Little is known about the stigmatizing attitudes toward people with GAD among Syrian students. The questionnaires contained demographic data about age, gender, social status, personal stigma toward GAD scale, perceived stigma toward GAD scale, social distance with those with GAD, the participants' usual source of their knowledge about GAD, helpful interventions, and supporting information. A total of 1,370 replies were collected, but only 1,358 were used for analysis as 12 participants declined to complete the survey. About 44.1% of participants agreed that people with GAD could snap out of the problem, most of them being females (32.4% of the total population). Compared to medical students, more non-medical students (7.1% of the total population) believed that anxiety is a sign of personal weakness. This study demonstrated that Syrian college students showed a high level of stigmatizing and socially distancing attitudes toward people with GAD, particularly female and non-medical students.

## Introduction

*Generalized Anxiety Disorder* can be defined as excessive feelings of worrying, stress, restlessness, and anxiety about various things in daily life (1, 2). GAD is considered a chronic disabling mental illness whose patients are usually underdiagnosed and undertreated (3). It is a highly prevalent disease (4), affecting about 1–5% of the general population (5) and about 10% of children and adolescents (6). The 12-month prevalence of GAD among US adults aged 18–64 years is about 2.9–3.1%, respectively; the lifetime prevalence in women is 7.7–4.6% in men (7). European study shows that GAD affects about 2% of adults in Europe and represents 10% of all mental disorders seen in primary care (8). About 25% of GAD cases develop at 20 years of age, and 50% of cases have an onset between 20 and 47 years (9). Studies show a high prevalence of GAD among students (10, 11), particularly those working in the healthcare field, such as physicians, dentists, and nurses (12–14). Although the exact etiology of GAD is still unknown, studies show that its etiology is multifactorial, including genetic, environmental, and biological factors such as the disturbance of neurotransmitters (15). People with GAD usually suffer from a mixture of psychological and biological symptoms like persistent worrying and restlessness with difficulty in trolling this feeling or identifying the source of it, fatigue, sleep disturbance, concentration difficulties, muscle tension, and aches, GIT unexplained pains, and tachycardia (1, 7). Studies showed that individuals with GAD reported less satisfaction with their quality of life than non-anxious adults (16), with a higher risk of suicide (17). In addition, Students suffering from GAD also show an increased risk of suicide ideation, especially medical students (18). In cardiac and diabetic patients and those with pulmonary or neurological disease, GAD is associated with a higher risk of complications and lower adherence to treatment, indicating that GAD has a catastrophic impact on other medical conditions (19). These reasons explain the high mortality rate among GAD patients (20). GAD, like other mental illnesses, is associated with stigmatizing attitudes. Patients with GAD, either alone or comorbid with depression, have reported greater levels of self-stigma (21). Recent studies show a high prevalence of stigmatizing attitudes and discrimination among adolescents; Participants also said that levels of perceived stigma are higher than personal stigma (22). The literal meaning of the word “Stigma” is a scar but is now known as a mark of shame. In other words, when people stigmatize someone, they shame them and reject or exclude them from the social public due to abnormal behaviors or characteristics (23). When we talk about mental illness in general, we can define *stigma* as a negative social attitude toward mentally ill people resulting from a misconception about the true nature of mental illness (23, 24). Like any other mentally ill patient, people with GAD suffer from a double war; first, they fight against their chronic worrying and restlessness, which affect them in a bad manner both psychologically and physiologically. Second, they fight against discriminative and stigmatizing attitudes from the general public. These attitudes make them feel shy and lonely, causing them to isolate themselves away from others. Self-stigma is the sensation of shame that a mentally ill patient has about himself/herself. At the same time, public stigma is the disparaging attitude of the general public toward those with mental illness (25). Studies have reported high stigmatizing attitudes among the population toward those with mental illness (26). The general public stigmatizes attitudes such as avoidance, isolation, and hostile behaviors arising from their beliefs that those with mental illness are dangerous and responsible for their behaviors (27). Stigma is highly prevalent, with most individuals with mental illness having reported stigma (28), not only those who are affected but also who are using services for mental health reasons, as shown by a Canadian study (27), WHO reported catastrophic effects of stigmatizing attitudes on family relationships and social exclusion (29). In addition to the previously mentioned dangerous consequences of stigma, another severe outcome is the patient's unwillingness to seek help. Studies show that about 70% of all mentally ill people receive no treatment due to their sensation of embarrassment from how others see them if they seek such help; this, in turn, causes their illness to deteriorate, which consequently affects their daily lives (30). As mentioned before, in addition to other mental illnesses, GAD is associated with a high mortality rate among affected people compared to other populations (31). Anti-stigma initiatives are held nowadays to face the stigma toward mental illness by correcting social concepts and spreading awareness about the danger of stigmatizing attitudes toward people and society (32).

Our study was designed to assess the prevalence of public stigma toward GAD, including personal and perceived stigma in a sample of Syrian students, and determine the reasons for these stigmata. We believe there are no current Syrian studies on this topic, and we do not have any previous data regarding the student population's GAD personal or perceived stigma. Therefore, we are holding this study to determine whether stigma is prevalent among Syrian students and assess the degree of stigmatizing attitudes and social distancing.

## Methods

The study was a cross-sectional study. Data were collected from the Online Google Form published on social media from 18 th to 27 th March 2021. We have distributed the form to trusted social media groups to avoid invalid or incomplete data due to randomization. Inclusion criteria involve Syrian students aged 18 years or above, and respondents should be in studying status.

We attempted to distribute the online survey in all Syrian governorates (Damascus, Rif Dimashq, Aleppo, Daraa, Deir ez-Zor, Hama, AlHasakah, Qamishli, Homs, Idlib, Latakia, Quneitra, ArRaqqah, As-Suwayda, Tartous) in order to get representative findings for the whole population (Syrian University students). “https;//www.calculator.net/sample-size-calculator.html” was used to calculate the sample size. According to latest data from “manhom.com” website, the estimated Syrian University students was 671580 students in 2017. The required sample size appeared to be 384 participants. The sample size was calculated as the population proportion to be 50%, at the 95% confidence level with a 5% marginal error. The total of participants who completed the online questionnaire was 1358 participants, with 12 participants who refused to complete it.

To verify accuracy and that all respondents could comprehend the survey, 30 people were given the task of filling it out. After that, a pilot test with 50 participants was conducted to confirm the validity and reliability of the questionnaire. Cronbach's alpha values for the regions varied from 0.70 to 0.80, showing that the tools maintained outstanding internal consistency. To get the required data from the respondents, we employed the convenience and snowball strategies.

### Measurements in the survey questionnaires

The questionnaires consisted of seven parts: the first part was a range of questions about demographic data like age, gender, and social status, the second part was questioned about personal stigma toward the GAD scale, and the third part included questions about perceived stigma toward GAD scale, the fourth part was closed-ended questions about social distance with those with GAD, the answers were Yes or No answers, the fifth part consists of questions about the participants' usual source of their knowledge about GAD like newspapers, TV or websites, the sixth part is concerned by helpfulness or intervention, this part is subdivided into four subgroups of a question with multiple answers, these subgroups are People who can help, Medications which can help, other interventions and help methods, the last part is concerned about supporting information, it includes three cases participants should answer to confirm their knowledge toward GAD in comparison to other mental illness.

### GAD stigma scale

The GAD Stigma Scale contains personal stigma subscales (nine items) and perceived stigma subscales (nine items). The statements in each item of the two subscales are the same except for the subject of items. In the personal stigma subscales, respondents were asked about their attitude toward people with GAD symptoms described in the vignette (e.g., “People with GAD could snap out of it if they wanted”). In the perceived stigma subscales, respondents were asked their beliefs about most of the other people's attitudes toward people with GAD symptoms described in the vignette (e.g., “Most people believe that people with GAD could snap out of it if they wanted”). The response of each item was measured on a five-point scale ranging from “strongly agree” to “strongly disagree” (1). The Chinese scale has been widely used with good reliability and validity (2).

### Social distance scale

The five-item short measurement of SDS was developed by (3) to measure the desire for social distancing from a person with mental illness. The Chinese version of the SDS was used to estimate the willingness to come into contact (such as live next door, marry into the family) with the person described in the vignette. The response of each item was measured on a four-point scale, which ranged from “definitely willing” to “definitely unwilling.” The reliability and validity of its Chinese version have been tested, and the results showed that all the indicators met the requirements of psychometrics.

### Ethics statement

The ethics committee at Damascus University and Aleppo University approved the protocol in March 2021. The convenience sampling method was used in the present study. Considering the representativeness of the sample, this study randomly selected different classes by school, grade, and study major. The aim of the present study was explained in the questionnaires, and informed consent was obtained from all the respondents through a Yes or No question inside the questionnaire asking participants whether they agreed to answer this questionnaire or not. They were encouraged to independently analyze the vignette and answer several questions, including demographic information, GAD Stigma Scale, and social distance scale SDS. The survey contained a cover page stating that responses were anonymous and voluntary and would not impact the participants.

### Statistical analysis

All data were analyzed by SPSS 22 and Excel. Descriptive statistics were applied for demographic data (percentage), stigma attitudes toward people with GAD (percentage frequencies and 95% CI), and social distance (percentage frequencies and 95% CI). The options of “agree” and “strongly agree” were combined into one option on the GAD Stigma Scale, and the options of “Yes” and “No” were combined into one option on the SDS. The combined options represent the positive and negative attitudes of the respondents. Mann-Whitney U and Kruskal-Wallis test assessed the significant difference in each item on the GAD Stigma Scale and SDS among different demographic variables (gender, major, educational level, and school level) in the proportion of agreement. The value of p was set at <0.05 for statistical significance.

## Results

### Baseline characteristics

One thousand three hundred sixty-six responses have been distributed, with 12 participants refusing to complete the survey. Overall, 1,358 responses we included for analysis. As shown in Table 1, the mean age of participants was 22.8 years, with 70% being females. Most of the participants are medical students (69.1%). Around one-third of the students (32.0%) have a job besides their studies. About two-thirds of the participants (65.2%) had a positive history of mental health disease, but only 4.5% were under current psychological treatment.

**Table 1:** Baseline characteristics of the participants.

	***N = 1,358***	
	***n***	**%**
Age (Mean/SD)	22.8 ± 2.68	
**Gender**		
Male	407	30.0%
Female	951	70.0%
**Social status**		
Single	1,215	89.5%
Married	130	9.6%
Divorced	8	0.6%
Widower	5	0.4%
**Major section**		
Medical student	938	69.1%
Non-medical student	420	30.9%
**Economic level**		
Bad	123	9.1%
Middle	845	62.2%
Good	353	26.0%
high	37	2.7%
**The University stage**		
1st year	203	14.9%
2nd year	267	19.7%
3rd year	224	16.5%
4th year	348	25.6%
5th year	155	11.4%
6th year	161	11.9%
**Region**		
City	1,027	75.6%
Rural	331	24.4%
**Occupation status**		
Worker	435	32.0%
Non-Worker	923	68.0%
**Live with**		
Family	1,143	84.2%
With father	16	1.2%
With mother	85	6.3%
With friends	114	8.4%
**Immigrant status**		
Yes	537	39.5%
No	821	60.5%
**History of mental health disease**		
Yes	886	65.2%
No	472	34.8%
**Current psychological treatment**		
Yes	61	4.5%
No	1,297	95.5%
**Current pharmacological treatment**		
Yes	161	11.9%
No	1,197	88.1%

### Personal stigma

Table 2 shows the differences between genders and medical and non-medical majors regarding the stigma attitudes toward the person with GAD. More females agreed that “The person could snap out of the problem” compared to males (32.4 vs. 12% of the total population, *p* < 0.05). More females (10.5% of the total population) and non-medical students (7.1% of the total population) believed that anxiety is a sign of personal weakness, compared to males and medical students, respectively. About a quarter of the population agreed that “Anxiety is not a real medical illness” (22.7%), with over half of them being medical students (11.5%). Regarding severe stigmatization, 11.8% of participants thought people with anxiety are dangerous. Over a quarter of the participants (25.4%) believed that people with this anxiety disorder are unpredictable, most of them being females (19.1% of the total population) compared to males (6.5% of the total population). More than one-fifth of participants (21.4%) will not tell anyone if they had the problem, and 9.2% will not employ someone with this problem.

**Table 2:** Percentage of participants who “agree” or “strongly agree” with personal stigma toward GAD patient scale statements.

**Statement about personal belief**	**Total (*****N** =* **935)**		**Gender**				**Major section**				**Region**				**Economic level**								**Occupation status**			
	***n***	**%**	**Male** **(*n =* 407)**		**Female** **(*n =* 951)**		**Medical** **(*n =* 938)**		**Non-Medical** **(*n =* 420)**		**City** **(*n =* 1,027)**		**Rural region** **(*n =* 331)**		**Low** **(*n =* 123)**		**Moderate** **(*n =* 845)**		**Good** **(***n =* **353)**		**High** **(*n =* 37)**		**Worker** **(*n =* 435)**		**No*****n-*****worker** **(*n =* 923)**	
			***n***	**% (95% CI)**	***n***	**% (95% CI)**	***n***	**% (95% CI)**	***n***	**% (95% CI)**	***n***	**% (95% CI)**	***n***	**% (95% CI)**	***n***	**% (95% CI)**	***n***	**% (95% CI)**	***n***	**% (95% CI)**	***n***	**% (95% CI)**	***n***	**% (95% CI)**	***n***	**% (95% CI)**
**The person could snap out of the problem**	**599**	**44.10%**	**162**	**12.00%**	**437**	32.4%**	**416**	**30.80%**	**183**	**13.6%**	**459**	**34.00%**	**140**	**10.40%**	**49**	**3.60%**	**373**	**27.70%**	**164**	**12.20%**	**13**	1%*	**203**	**15.00%**	**396**	**29.40%**
**Problem is a sign of personal weakness**	**218**	**16.10%**	**77**	**5.70%**	**141**	**10.5%**	**123**	**9.10%**	**95**	7.1%**	**154**	**11.40%**	**64**	4.8%*	**25**	**1.90%**	**141**	**10.50%**	**46**	**3.40%**	**6**	**0.40%**	**73**	**5.40%**	**145**	**10.80%**
**Problem is not a real medical illness**	**308**	**22.70%**	**96**	**7.10%**	**212**	**15.8%**	**155**	**11.50%**	**153**	11.4%**	**211**	**15.70%**	**97**	7.2%*	**29**	**2.20%**	**202**	**15.00%**	**70**	**5.20%**	**7**	0.5%*	**100**	**7.40%**	**208**	**15.50%**
**People with this problem are dangerous**	**160**	**11.80%**	**62**	**4.60%**	**98**	7.30%*	**112**	**8.30%**	**48**	**3.60%**	**115**	**8.60%**	**45**	**3.40%**	**23**	**1.70%**	**106**	**7.90%**	**28**	**2.10%**	**3**	**0.20%**	**39**	**2.90%**	**121**	**9.00%**
**Avoid people with this problem**	**323**	**23.80%**	**87**	**6.50%**	**236**	**17.60%**	**199**	**14.80%**	**124**	9.2%**	**239**	**17.80%**	**84**	**6.20%**	**35**	**2.60%**	**201**	**15.00%**	**76**	**5.70%**	**11**	**0.80%**	**94**	**7.00%**	**229**	**17.00%**
**People with this problem are unpredictable**	**345**	**25.40%**	**88**	**6.50%**	**257**	19.1%*	**228**	**17.00%**	**117**	**8.70%**	**270**	**20.10%**	**75**	**5.60%**	**42**	**3.10%**	**202**	**15.00%**	**91**	**6.80%**	**10**	0.7%*	**110**	**8.20%**	**235**	17.5%*
**If I had this problem, I would not tell anyone**	**291**	**21.40%**	**92**	**6.90%**	**199**	**14.90%**	**200**	**15.00%**	**91**	**6.8%**	**223**	**16.70%**	**68**	**5.10%**	**27**	**2.00%**	**167**	**12.50%**	**83**	**6.20%**	**14**	**1.00%**	**95**	**7.10%**	**196**	**14.70%**
**Would not employ someone with this problem**	**125**	**9.20%**	**52**	**3.90%**	**73**	5.40%*	**67**	**5.00%**	**58**	**4.30%**	**86**	**6.40%**	**39**	**2.90%**	**19**	**1.40%**	**73**	**5.40%**	**28**	**2.10%**	**5**	0.4%*	**47**	**3.50%**	**78**	**5.80%**
**Would not vote for a politician with this problem**	**160**	**11.80%**	**58**	**4.30%**	**102**	**7.60%**	**97**	**7.20%**	**63**	4.7%**	**112**	**8.30%**	**48**	**3.60%**	**20**	**1.50%**	**94**	**7.00%**	**35**	**2.60%**	**11**	**0.85**	**60**	**4.50%**	**100**	**7.40%**
**GPSS total score (mean ± SD)**	**1.8**	**1.5**	**1.9**	**1.6**	**1.8**	**1.5**	**1.7**	**1.4**	**2.2**	1.8*	**1.8**	**1.5**	**2**	**1.5**	**2.2**	**1.9**	**1.8**	**1.4**	**1.7**	**1.6**	**2.1**	**1.5**	**1.9**	**1.6**	**1.8**	**1.5**

CI, confidence interval; GPSS, GAD personal stigma scale. Data are n, %, or mean ± SD. *p < 0.05.

### Perceived stigma

Differences between genders and medical and non-medical majors regarding the percentage of participants agreeing that others may have stigma attitudes toward people with GAD are shown in Table 3. Over half the participants (50.9%) thought that “Most people believe that people with anxiety could snap out of it if they wanted,” more than half of them being females (39.9% of the total population vs. 14.6%, *p* < 0.05). More than half of the participants believed that other people would not consider anxiety a medical illness; compared to personal stigma (Table 2), less than a quarter of participants (22.7%) had that as a personal belief. Around 39.2% of participants believed that “If they had anxiety, most people would not tell anyone,” with 32.9% medical students compared to 10.3% non-medical students. Similarly, 39.1% of participants agreed that “Most people would not employ someone they knew had been affected with anxiety,” with the majority being females (30.4% of the total population) compared to 12.8% being males.

**Table 3:** Percentage of participants who “agree” or “strongly agree” with perceived stigma toward GAD patient scale statements.

**Statement about perceived belief**	**Total (** ***N =* 1,358)**		**Gender**				**Major section**				**Region**				**Economic level**								**Occupation status**			
	***n***	**%**	**Male** **(*n =* 407)**		**Female** **(*n =* 951)**		**Medical** **(*n =* 938)**		**Non-Medical** **(*n =* 420)**		**City** **(*n =* 1027)**		**Rural region** **(*n =* 331)**		**Low** **(*n =* 123)**		**Moderate** **(*n =* 845)**		**Good** **(*n =* 353)**		**High** **(*n =* 37)**		**Worker** **(*n =* 435)**		**Non*****-*****worker** **(*n =* 923)**	
			***n***	**% (95% CI)**	***n***	**% (95% CI)**	***n***	**% (95% CI)**	***n***	**% (95% CI)**	***n***	**% (95% CI)**	***n***	**% (95% CI)**	***n***	**% (95% CI)**	***n***	**% (95% CI)**	***n***	**% (95% CI)**	***n***	**% (95% CI)**	***n***	**% (95% CI)**	***n***	**% (95% CI)**
**Most people believe that people with depression could snap out of it if they wanted**	**691**	**50.90%**	**185**	**14.60%**	**506**	39.9%**	**485**	**38.20%**	**206**	**16.20%**	**538**	**42.40%**	**153**	**12.10%**	**58**	**4.60%**	**431**	**34.00%**	**176**	**13.90%**	**26**	**2.10%**	**222**	**17.50%**	**469**	**37.00%**
**Most people believe that anxiety is a sign of personal weakness**.	**620**	**45.70%**	**185**	**14.80%**	**435**	**34.7%**	**443**	**35.30%**	**177**	**14.1%**	**459**	**36.60%**	**161**	**12.8%**	**59**	**4.70%**	**380**	**30.30%**	**157**	**12.50%**	**24**	**13.90%**	**198**	**15.80%**	**422**	**33.70%**
**Most people believe that anxiety is not a medical illness**.	**697**	**51.30%**	**198**	**16.00%**	**499**	**40.3%**	**499**	**40.30%**	**198**	**16%**	**520**	**42.00%**	**177**	**14.30%**	**67**	**5.40%**	**430**	**34.70%**	**174**	**14.10%**	**26**	**2.10%**	**227**	**18.30%**	**470**	**38.00%**
**Most people believe that people with anxiety are dangerous**.	**371**	**27.30%**	**106**	**8.60%**	**265**	**21.5%**	**280**	**22.70%**	**91**	7.4%**	**262**	**21.20%**	**109**	8.80%*	**40**	**3.20%**	**221**	**17.90%**	**99**	**8.00%**	**11**	**0.90%**	**124**	**10.10%**	**247**	**20.00%**
**Most people believe that it is best to avoid people with anxiety so that you don't become depressed yourself**.	**594**	**43.70%**	**167**	**13.50%**	**427**	**34.6%**	**419**	**34.00%**	**175**	**14.2%**	**438**	**35.50%**	**156**	**12.6%**	**61**	**4.90%**	**365**	**29.60%**	**148**	**12.00%**	**20**	**1.60%**	**189**	**15.30%**	**405**	**32.80%**
**Most people believe that people with anxiety are unpredictable**.	**391**	**28.80%**	**90**	**7.30%**	**301**	24.5%*	**227**	**22.50%**	**114**	9.3%**	**302**	**24.50%**	**89**	**7.20%**	**63**	**2.90%**	**254**	**20.60%**	**88**	**7.10%**	**13**	**1.10%**	**124**	**10.10%**	**267**	**21.70%**
**If they had anxiety most people would not tell anyone**.	**532**	**39.20%**	**157**	**12.80%**	**375**	**30.5%**	**405**	**32.90%**	**127**	**10.3%**	**402**	**32.70%**	**130**	**10.60%**	**50**	**4.10%**	**339**	**27.60%**	**128**	**10.40%**	**15**	**1.20%**	**164**	**13.30%**	**368**	**29.90%**
**Most people would not employ someone they knew had been affected with anxiety**	**531**	**39.10%**	**157**	**12.80%**	**374**	**30.4%**	**383**	**31.10%**	**148**	**12%**	**383**	**31.10%**	**148**	**12%**	**54**	**4.40%**	**324**	**26.30%**	**135**	**11.00%**	**18**	**1.50%**	**168**	**13.70%**	**363**	**29.50%**
**Most people would not vote for a politician they knew had been affected with anxiety**	**464**	**34.20%**	**142**	**11.50%**	**322**	**26.1%**	**343**	**27.80%**	**121**	9.8%*	**344**	**27.90%**	**120**	**9.70%**	**40**	**3.20%**	**291**	**23.60%**	**116**	**9.40%**	**17**	**1.40%**	**150**	**12.20%**	**314**	**25.40%**
**GPSS total score (mean ± SD)**	**3.96**	**2.7**	**3.6**	**2.6**	**4**	2.8**	**4.1**	**2.7**	**3.6**	2.6**	**3.9**	**2.7**	**4.2**	**2.6**	**4.3**	**2.4**	**3.9**	**2.7**	**3.8**	**2.8**	**4.7**	**3**	**3.9**	**2.8**	**3.9**	**2.7**

CI, confidence interval; GPSS, GAD perceived stigma scale. Data are n, %, or mean ± SD. *p < 0.05.

### Social distance

Table 4 shows the participants' willingness to socialize with people with GAD. Over half the participants (51.6%) are not willing to live next to a person with GAD, with female students being more unwilling to do so (35% of the total population vs. 17.5%, *p* < 0.05). Economic level and working status were also significantly associated with unwillingness to live next to GAD individuals. In addition, the vast majority of participants (91.9%) will not marry into a family with GAD individuals, particularly the female participants (64.8% of the general population vs. 28.2%, *p* < 0.05). Non-medical students were significantly more willing to make friends with GAD than medical students (14.4% of the general population vs. 27.3%, *p* < 0.05).

**Table 4:** Percentage of participants who are “probably unwilling” or “definitely unwilling” to have contact with GAD patient.

**Statement about personal belief (SDS)**	**Total (** ***N =* 1,358)**		**Gender**				**Major section**				**Region**				**Economic level**								**Occupation status**			
	***n***	**%**	**Male** **(*n =* 407)**		**Female** **(*n =* 951)**		**Medical** **(*n =* 938)**		**Non-medical** **(*n =* 420)**		**City** **(*n =* 1,027)**		**Rural region** **(*n =* 331)**		**Low** **(*n =* 123)**		**Moderate** **(*n =* 845)**		**Good** **(*n =* 353)**		**High** **(*n =* 37)**		**Worker** **(*n =* 435)**		**Non-worker** **(*n =* 923)**	
			***n***	**%**	***n***	**%**	***n***	**%**	***n***	**%**	***n***	**%**	***n***	**%**	***n***	**%**	***n***	**%**	***n***	**%**	***n***	**%**	***n***	**%**	***n***	**%**
**Live next door**	**701**	**51.60%**	**234**	**17.50%**	**467**	35%*	**480**	**35.90%**	**221**	**16.50%**	**530**	**39.70%**	**171**	**12.80%**	**60**	**4.50%**	**428**	**32.00%**	**200**	**15.00%**	**13**	1%*	**493**	**36.90%**	**208**	15.6%*
**Spend the evening socializing**	**609**	**44.80%**	**169**	**12.60%**	**440**	**32.80%**	**431**	**32.10%**	**178**	**13.30%**	**465**	**34.60%**	**144**	**10.70%**	**46**	**3.40%**	**371**	**27.60%**	**179**	**13.30%**	**13**	1%*	**407**	**30.30%**	**202**	**15.1%**
**Make friends**	**560**	**41.20%**	**163**	**12.10%**	**397**	**29.50%**	**367**	**27.30%**	**193**	14.4%*	**427**	**31.80%**	**133**	**9.90%**	**51**	**3.80%**	**345**	**25.70%**	**146**	**10.90%**	**18**	**1.30%**	**395**	**29.40%**	**165**	**12.3%**
**Work closely**	**734**	**54.10%**	**230**	**17.20%**	**504**	**37.70%**	**493**	**36.90%**	**241**	**18.00%**	**567**	**42.40%**	**167**	**12.50%**	**58**	**4.30%**	**456**	**34.10%**	**204**	**15.30%**	**16**	**1.20%**	**520**	**38.90%**	**214**	16%*
**Marry into family**	**1,248**	**91.90%**	**379**	**28.20%**	**869**	64.8%*	**864**	**64.40%**	**384**	**28.60%**	**947**	**70.60%**	**301**	**22.40%**	**107**	**8.00%**	**777**	**57.90%**	**332**	**24.70%**	**32**	2.4%*	**853**	**63.60%**	**395**	**29.4%**
**GSS total scoremean ± SD)**	**2.88**	**1.40%**	**2.9513**	**1.41%**	**2.85**	**1.40%**	**2.84**	**1.40%**	**2.96**	1.41%*	**2.9026**	**1.39%**	**2.81**	**1.43%**	**2.6923**	**1.42%**	**2.84**	**1.40%**	**3.071**	**1.35%**	**2.52**	**1.61%**	**2.94**	**1.39%**	**2.7536**	1.43%*

CI, confidence interval; SDS, social distance scale. Data are n, %, or mean ± SD. *p < 0.05.

Table 5 shows the predictors for stigma and social distance. These predictors include gender, age, major study selection, economic level, region settings, and occupation status. We used multiple linear regression to analyze this relationship.

**Table 5:** Predictors for stigma and social distance by multiple linear regression analysis.

**Dependent variable predictors**	***B***	***t***	P^*^	***R***	***R* ^2^**	**Adj.*R*^2^**
**GPSS**				0.175	0.31	0.26
Gender	0.069	0.720	0.472			
Age	0.002	1.097	0.273			
Major section	0.569	5.952	0.000			
Economic level	0.041	0.604	0.546			
Region settings	0.188	1.859	0.063			
Occupation status	0.096	1.003	0.316			
GPSS*				0.128	0.16	0.011
Gender	0.487	2.763	0.006			
Age	0.016	0.790	0.429			
Major section	0.500	2.713	0.007			
Economic level	0.065	0.512	0.609			
Region settings	0.333	1.774	0.076			
Occupation status	0.208	1.159	0.247			
**SDS**				0.113	0.013	0.0008
Gender	0.169	1.953	0.051			
Age	0.000	0.115	0.909			
Major section	0.185	2.143	0.032			
Economic level	0.123	1.995	0.046			
Region settings	0.078	0.856	0.392			
Occupation status	0.243	2.823	0.005			

GPSS, GAD personal stigma scale; GPSS*, GAD perceived stigma scale.

### Sources of information

**Figure 1:**
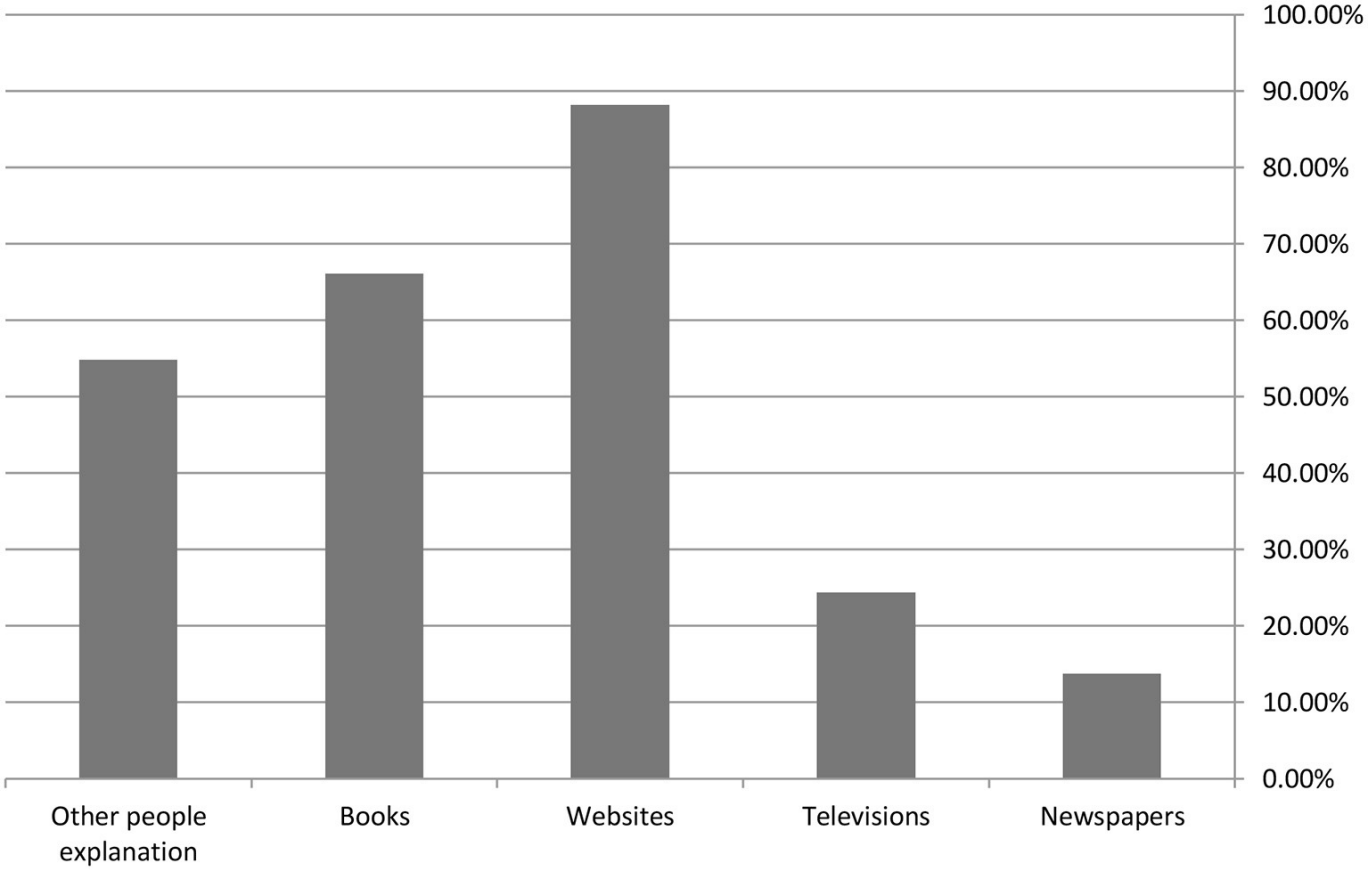
Sources of information that the participants used to learn about mental health.

Regarding the sources of information that the participants used to learn about mental health, as shown in Figure 1, websites were the most commonly used source (88.2%), followed by books (66.1%), other people's explanations (54.8%), television (24.4%) and newspapers (13.8%).

### People who can help

Regarding people the participants thought they could help, a psychiatrist was their top choice (79.5%), as shown in Table 6. This was followed by a friend (65.9%), a close family member (64.1%), and praying to god. The least practical choice was an herbalist (6%).

**Table 6:** Helpful interventions.

	**Number**	**Percentage**
**People who can help**		
A typical GP or family doctor	751	55.5%
A pharmacist	222	16.4%
A counselor	345	25.5%
A social worker	354	26.2%
A telephone counseling service	82	6.1%
A psychiatrist	1,075	79.5%
A psychiatric nurse	380	28.1%
A clinical psychologist	682	50.4%
Help from close family	866	64.1%
Help from close friends	891	65.9%
An herbalist	81	6%
Pray to god for help	790	58.4%
**Medication that can help**		
Vitamins and mineral	557	43.6%
Laxatives such as lactulose or Senna	27	2.1%
Tonics or herbal medicines	193	15.1%
Antibiotics	58	4.5%
Antidepressants	687	53.8%
Pain relievers such as aspirin or acetaminophen	128	10%
Sleeping pills	198	15.5%
Antipsychotics	154	12.1%
Tranquilizer such as diazepam	421	32.9%
Anxiolytics	1,005	78.6%
**Other Inventions**		
Becoming physically more active, such as playing more sports, or doing a lot more walking or gardening.	1,103	82.9%
Undergoing electro-convulsive therapy.	32	2.4%
Getting out more.	769	57.8%
Staying at home and resting.	235	17.7%
Having an occasional alcoholic drink to relax.	62	4.7%
Psychotherapy	850	63.9%
Attending courses on relaxation, stress management, meditation, or yoga	557	41.8%
Cutting out alcohol altogether.	549	41.2%
Massage to relax.	419	31.5%
Acupuncture therapy.	54	4.1%
Being admitted to a psychiatric hospital.	82	6.2%
Reading about people with similar problems and how they have dealt with them.	790	59.4%
Going on a special diet or avoiding certain foods.	387	29.1%
Aromatic therapy.	125	9.4%
Hypnosis	95	7.1%
Being admitted to a psychiatric ward or general hospital.	82	6.2%
**Help methods**		
Encourage the person to seek help.	784	59.5%
Accompany the person to professional help.	658	50%
Contact professional help on the person‘s behalf.	128	9.7%
Listen with the person	768	58.3%
Encourage the person to see a community physician.	368	27.9%
Encourage the person to see a counselor.	287	21.8%
Encourage the person to see psychiatrist.	857	65.1%
Give advice.	731	55.5%
Encourage the person to go to hospital.	124	9.4%
Encourage the person to see psychologist.	657	49.9%
Encourage the person to go to a mental health clinic.	168	12.8%
Ask if the person wants help	658	50%
Assess the problem/risk of harm.	303	23%
Do an intervention.	127	9.6%
Cheer the person up/boost the person‘s confidence.	797	60.5%
Tell the person‘s parents or family.	347	26.3%
Seek information for the person.	573	43.5%
Help the person make new friends.	600	45.6%
Help with chores/work.	313	23.8%
Provide general support (e.g., practical emotional).	637	48.4%
Spend time/socialize with the person.	653	49.6%
Encourage the person to become physically active.	746	56.6%

### Medications

Anxiolytics were the most chosen medications (78.6%), as shown in Table 6. Antidepressants (53.8%), vitamins and minerals (43.6%), and tranquilizers (32.9%) came next.

### Other interventions

Most participants (82.9%) agreed that becoming more physically fit positively impacts the people suffering from GAD. Psychotherapy came second (63.9%), followed by reading about people with similar problems and how they have dealt with them (59.4%) and getting out more (57.8%), as shown in Table 6. Electroconvulsive therapy was believed to be the least helpful intervention (2.4%).

### Help methods

Many help methods were believed to be helpful. As shown in Table 5, the most agreed-upon method encouraged the person to see a psychiatrist (65.1%). Furthermore, over half the participants chose to cheer the person up (60.5%), encourage the person to seek help (59.5%), listen to the person (58.3%), and encourage the person to become physically active (56.6%), and giving advice (55.5%). The least chosen method was encouraging the person to go to the hospital (9.4%).

### Knowledge toward three mental disorders

**Figure 2:**
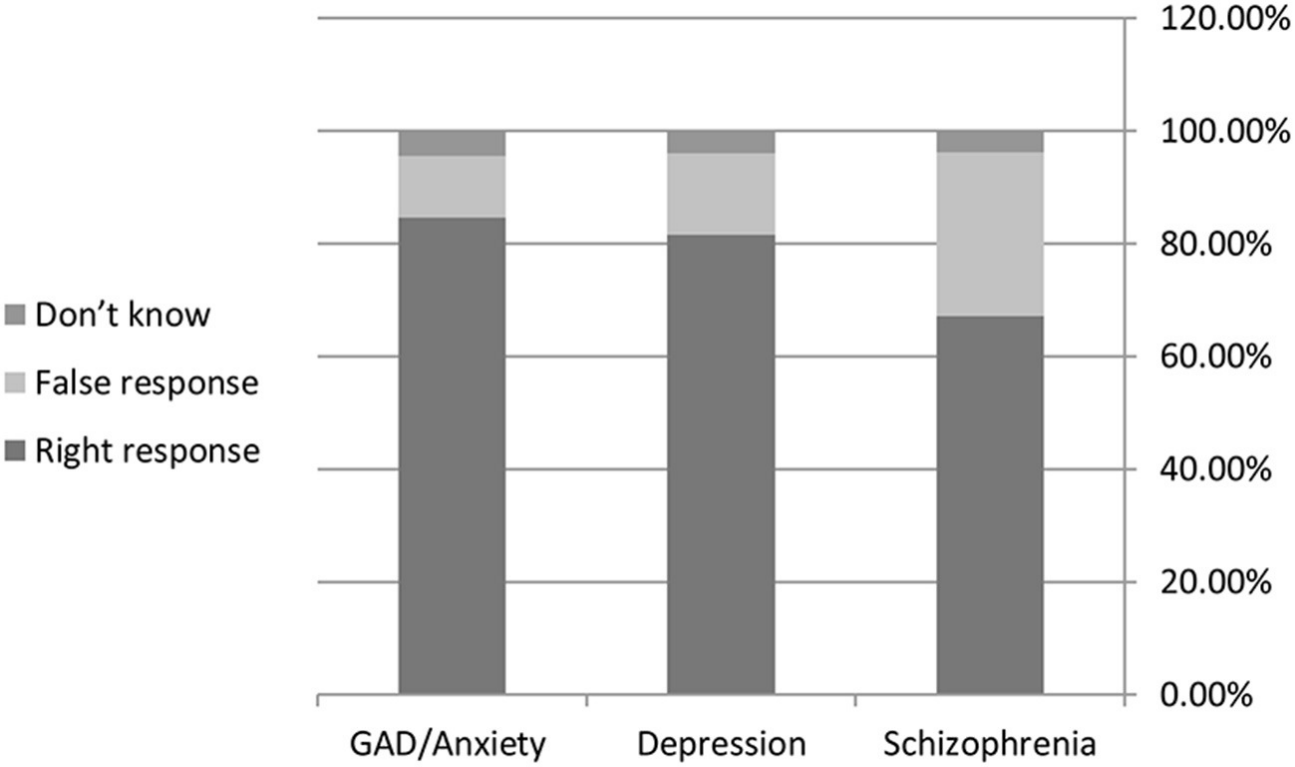
Supporting information: (Knowledge toward the three mental disorders).

As shown in Figure 2, anxiety was the most well-known mental disorder among the participants, with 84.7% reaching the correct diagnosis of the vignette. This was followed by depression, then Schizophrenia, with 81.6% and 67.1% of students reaching the correct answer.

## Discussion

Mental health disorders continue to pose a threat to the minds of people. From how it seems, it is apparent that these disorders pose a risk to people's lives nearly equal to or even more than the one posed by physical disorders or diseases. An added burden of mental health disorders is added to the lives of the patient because of the major fact that most people usually delay in seeking professional help from psychiatrists (33).

Generalized Anxiety Disorder, or GAD for short, is another mental health disorder that comes with a myriad of confusing symptoms that are enough to make a person feel overwhelmed due to stress (20). However, a much-feared complication regarding GAD is anticipated when it gets complicated due to the addition of stigma (34).

Now, this stigma has become a more significant part of the problem. It complicates the severity and intensity of Generalized Anxiety Disorder (GAD) but also the treatment-seeking and therapy process (35). The patients feel reluctant to seek treatment out of their rational and irrational fears, and if nothing, this whole thing delays the entire process that could have otherwise helped the patient had they gone to seek treatment for it (36).

Therefore, this study was carried out to elaborate and elucidate the causes of GAD and its stigmas. The target population was centered explicitly around students from both medical and non-medical fields to assess whether their current knowledge regarding the topic was sufficient to understand the condition and the stigmas attached to it.

The responses were collected from a set target population of 1,366 participants. These comprised males and females, with the latter dominating the questionnaire population. Students who worked part-time and their studies were also included in the study. The purpose was to include students from that stage of life where they were especially prone to developing anxiety and related disorders. Interestingly enough, out of the selected total target population, most of the females (32.4%) seemed to acknowledge the existence of anxiety. They also acknowledged that coming out of this 'zone' was easy as a snap and that anxiety denotes a personal weakness present within the individual themselves. Coincidentally, the females who favored this idea were medical students. This establishes that medical students know the presence of such conditions and know they can develop them due to their work and studies. The normalization of this fact is a step toward ending the stigma. Although it will take time to make the masses aware of this deal, it is plausible to see that the younger generations seem to be fine with the idea and understand its dynamics. However, 21.4% of the target population was among those people who refused to admit whether they had ever felt that they suffered from these issues or illnesses or not. This fact, again, points out that it will take time to end the stigma.

Compared to this study, another similar study was carried out among the first-year psychology students of the University of Canberra regarding the role of certain variables on their personal, perceived self-stigmatization of widespread mental health disorders such as anxiety and depression. The study was carried out on online questionnaires, with the reward of research hours in return. Toward the end of the study, it was concluded that since these students were highly-aware of the dynamics of both these illnesses, they had significantly lower personal stigmas for either of these conditions. Overall, this study helped promote that increased planned campaigning toward reducing stigmas plays a more significant role in eliminating the presence of social stigmas regarding these mental health disorders (37). Stigmas have always posed significant challenges and risks to the population that is considered the target audience here: people suffering from GAD. Stigmas bring forth severe economic, physiological, and psychological consequences (24, 38). It is undoubtedly due to these social and personal barriers that people consider it taboo to seek help for their issues and disorders (39). The behavioral impacts that occur due to these stigmas are widely distributed into different types (40). These include avoiding help, withholding feelings, isolating oneself from the world, and a complete reluctance to seek treatment (41). As a result, the affected people were also more inclined toward developing negative behaviors such as low self-esteem, lower adherence to no plans of seeking treatment, early withdrawal from therapies, and general secretive nature (42).

Several factors are associated with developing different types of stigma in such people. The findings are consistent with the reality that more people need to be brought forward toward accepting the presence of an illness in themselves, which would ultimately lead them toward another step ahead, which would be the step toward taking treatments for their diagnosed conditions (43). It will indeed be a positive approach as more people start to come to therapy. For this purpose, awareness campaigns are thought to be the perfect way to educate the masses regarding the implications of not seeking therapy and making people aware that they need to come toward this side of the picture (44).

As far as the implications of this study are concerned, it is undoubtedly the limited target population. Although the study was specifically designed to target students, it is equally important to question and study the people who directly impact the lives of these students, such as their parents and teachers. These people have a more significant impact than they are given credit for, so getting to know their stance regarding the whole situation will only add to the broadness of this literature and help in learning more regarding the level of knowledge and perception that older populations have regarding such illnesses. A bright perspective can be viewed in the light that teachers and parents can serve as a perfect comparative group for studying their opinions and knowledge regarding these mental health disorders.

Moreover, because the patients refuse to admit that something is wrong with them, social isolation has also led people to falsely mark their answers when asked on research questionnaires to pose as if everything is all right in their lives. This further implicates the awareness process.

We recommend that more social campaigns can clear some misconceptions about GAD. Local medical schools should also invest more effort in teaching about psychological disorders in general and GAD in particular. Judging by the study results, it may take a long time to correct this stigmatization. Thus, implanting precise and true definitions of GAD in pre-college schools should be helpful. Lastly, we emphasize the importance of establishing a safe environment for GAD patients to seek help with full encouragement.

## Limitations

The data collection process was done through online google forms. So, we were unable to prevent some biased or unorganized answers. We have tried to minimize the partial data by distributing the survey equally among all Syrian counties. Also, female respondents formed more than two-thirds of the sample data. Furthermore, we declared that there is no previous study that assessed the validity of the used tools in our study in arab countries.

## Conclusion

We observed a high level of stigmatizing and socially distancing attitudes toward people with GAD. Female and non-medical students had more levels than male and medical students. Even more significantly, most participants thought that other people might highly mistreat and stigmatize GAD people. Syrian students have a substantial GAD stigma, whether from a medical or non-medical background. We suggest that more studies be held to determine if this stigma still exists in other groups of the Syrian community. We advise that it is also important to invest in a more effective social campaign to help eliminate this stigma.

## Data availability statement

The original contributions presented in the study are included in the article/supplementary files, further inquiries can be directed to the corresponding author.

## Ethics statement

The studies involving human participants were reviewed and approved by the Ethics Committees in Damascus University and Aleppo University. The patients/participants provided their written informed consent to participate in this study.

## Author contributions

SSw took the lead in organizing and writing the manuscript. All authors contributed to the article and approved the submitted version.

## Conflict of interest

The authors declare that the research was conducted in the absence of any commercial or financial relationships that could be construed as a potential conflict of interest.

## Publisher's note

All claims expressed in this article are solely those of the authors and do not necessarily represent those of their affiliated organizations, or those of the publisher, the editors and the reviewers. Any product that may be evaluated in this article, or claim that may be made by its manufacturer, is not guaranteed or endorsed by the publisher.
