# Corrigendum: Attitude of Syrian students toward GAD patients: An online cross-sectional study

**DOI:** 10.3389/fpubh.2022.1112817

**Published:** 2022-12-16

**Authors:** Sarya Swed, Sheikh Shoib, Ubaid Khan, Amro A. El-Sakka, Mohammad Badr Almoshantaf, Noheir Ashraf Ibrahem Fathy Hassan, Lina Taha Khairy, Agyad Bakkour, Ali Hadi Hussein Muwaili, Karam R. Motawea, Fatima Abubaker Abdalla Abdelmajid, Eman Mohammed Sharif Ahmad, Safaa Mohamed Alsharief Ahmed, Mohammad Mehedi Hasan, Bisher Sawaf, Mhd Kutaiba Albuni, Elias Battikh, Asmaa Zainabo, Hidar Alibrahim, Hazem S. Ghaith, Nashaat Kamal Hamdy Elkalagi

**Affiliations:** ^1^Faculty of Medicine, Aleppo University, Aleppo, Syria; ^2^Department of Psychiatry, Jawahar Lal Nehru Memorial Hospital, Srinagar, Kashmir, India; ^3^Faculty of Medicine, King Edward Medical University Lahore, Lahore, Pakistan; ^4^Faculty of Medicine, Suez Canal University, Ismailia, Egypt; ^5^Department of Neurosurgery, Ibn Al-Nafees Hospital, Damascus, Syria; ^6^Faculty of Medicine, Aswan University, Aswan, Egypt; ^7^Faculty of Medicine, The National Ribat University, Khartoum, Sudan; ^8^Faculty of Medicine, Albaath University, Homs, Syria; ^9^Faculty of Medicine, Ivano-Frankivsk National Medical University, Ivano-Frankivsk, Ukraine; ^10^Faculty of Medicine, Alexandria University, Alexandria, Egypt; ^11^Faculty of Medicine, University of Medical Sciences and Technology, Khartoum, Sudan; ^12^Faculty of Medicine, Nile Valley University, Atbara, Sudan; ^13^Faculty of Medicine, Shendi University, Shendi, Sudan; ^14^Department of Biochemistry and Molecular Biology, Faculty of Life Science, Mawlana Bhashani Science and Technology University, Tangail, Bangladesh; ^15^Department of Internal Medicine, Syrian Private University, Damascus, Syria; ^16^Faculty of Medicine, Damascus University, Damascus, Syria; ^17^Faculty of Medicine, Tishreen University, Lattakia, Syria; ^18^Faculty of Medicine, Al-Azhar University, Cairo, Egypt; ^19^Lecturer in Internal Medicine and Tropical Medicine at Faculty of Medicine Al Arish University, Alarish, Egypt

**Keywords:** GAD (general anxiety disorders), stigma, national, Syria, students

In the published article, there was an error in affiliation 4. Instead of “Faculty of Veterinary Medicine, Suez Canal University, Ismailia, Egypt,” it should be “Faculty of Medicine, Suez Canal University, Ismailia, Egypt.”

In addition, an author's name was incorrectly displayed. Instead of Noheir A. I. F. Hassan it should be Noheir Ashraf Ibrahem Fathy Hassan.

The authors apologize for this error and state that this does not change the scientific conclusions of the article in any way. The original article has been updated.

